# Sparassis latifolia and exercise training as complementary medicine mitigated the 5-fluorouracil potent side effects in mice with colorectal cancer: bioinformatics approaches, novel monitoring pathological metrics, screening signatures, and innovative management tactic

**DOI:** 10.1186/s12935-024-03328-y

**Published:** 2024-04-18

**Authors:** Navid Abedpoor, Farzaneh Taghian, Khosro Jalali Dehkordi, Kamran Safavi

**Affiliations:** 1 Department of Sports Physiology, Faculty of Sports Sciences, School of Sports Sciences, Isfahan (Khorasgan) Branch, Islamic Azad University, Isfahan, Iran; 2 Department of Plant Biotechnology, Medicinal Plants Research Centre, Isfahan (Khorasgan) Branch, Islamic Azad University, Isfahan, Iran

**Keywords:** Colorectal Cancer, AOM, DSS, 5-Fluorouracil, Sparassis latifolia, Exercise, Biomarkers, Diagnosis, Palliative care

## Abstract

**Background:**

Prompt identification and assessment of the disease are essential for reducing the death rate associated with colorectal cancer (COL). Identifying specific causal or sensitive components, such as coding RNA (cRNA) and non-coding RNAs (ncRNAs), may greatly aid in the early detection of colorectal cancer.

**Methods:**

For this purpose, we gave natural chemicals obtained from Sparassis latifolia (SLPs) either alone or in conjunction with chemotherapy (5-Fluorouracil to a mouse colorectal tumor model induced by AOM-DSS. The transcription profile of non-coding RNAs (ncRNAs) and their target hub genes was evaluated using qPCR Real-Time, and ELISA techniques.

**Results:**

MSX2, MMP7, ITIH4, and COL1A2 were identified as factors in inflammation and oxidative stress, leading to the development of COL. The hub genes listed, upstream regulatory factors such as lncRNA PVT1, NEAT1, KCNQ1OT1, SNHG16, and miR-132-3p have been discovered as biomarkers for prognosis and diagnosis of COL. The SLPs and exercise, effectively decreased the size and quantity of tumors.

**Conclusions:**

This effect may be attributed to the modulation of gene expression levels, including MSX2, MMP7, ITIH4, COL1A2, PVT1, NEAT1, KCNQ1OT1, SNHG16, and miR-132-3p. Ultimately, SLPs and exercise have the capacity to be regarded as complementing and enhancing chemotherapy treatments, owing to their efficacious components.

## Introduction

The current status of Colorectal cancer (CRC) prevalence as the second most lethal form of cancer globally has shown a persistent upward trend [1]. Epidemiological research has indicated that most nations consider it a significant public health concern [2]. Moreover, it has been shown in recent research that the creation and progression of colorectal cancer (CRC) is regulated by cytokines associated with inflammation and the organization of extracellular structures [3, 4]. Growing research has revealed that inflammatory response pathways and extracellular matrix may play a pivotal role in developing microenvironmental CRC conditions [5]. Moreover, the cytokine storm condition destroyed the intestinal structure and extracellular matrix. Hence, there is a correlation between extracellular structure organization and inflammatory response pathways [6]. Stimulating the inflammation molecules produces the pro-inflammation molecules, which can promote the differentiation and proliferation of solid tumors.

In the clinical treatment of CRC, anti-inflammatory drugs are frequently employed alongside surgical resection [7, 8]. For a considerable duration, chemotherapy using 5-fluorouracil (5-FU) has been used as the primary therapeutic approach for individuals diagnosed with colorectal cancer (CRC) [9]. While medication treatment has shown efficacy in treating the majority of colorectal cancer (CRC) patients during the early stages, the subsequent development of drug resistance may lead to a negative prognosis for individuals with cancer [10]. Currently, 5-fluorouracil-based chemotherapy continues to be the primary treatment option for colorectal cancer (CRC). Based on the evidence, stage I and II patients had a 30% chance of recurrence following surgery within five years, whereas stage III patients had a 50–60% chance [11–13]. Thus, 5-FU regimens followed by surgery are the standard therapy for stage III and high-risk stage II CRC patients, significantly decreasing recurrence risk [14]. Most patients improve from chemotherapy, but others might not improve and die from adverse effects after numerous treatments [15, 16]. Drug resistance causes chemotherapeutic nonresponse in these individuals. On the other hand, despite chemotherapy efficacy, these drugs present potent side effects and are unsuitable for long-term use [17]. Hence, comprehending the underlying mechanisms of chemoresistance in CRC, enhancing the efficacy of existing treatment approaches, and discovering a potential functional component in the early stage of CRC is of utmost importance for preventing and controlling the disease [10].

Early diagnosis and prognosis are prerequisites for mitigating mortality in CRC patients. Some causative or susceptible elements, ceRNA network, detected in CRC are strongly associated with diagnostic approaches [18, 19]. Although several pathomechanisms influence CRC, critical mRNA-ncRNAs' precise role remains unclear [20].

Non-coding RNAs regulate gene transcription and expression as regulatory agents in biological processes. Thus, regulatory molecules may indicate disease prognosis, diagnosis, monitoring, and follow-up [4]. Differential expression of genes and non-coding RNAs may provide molecular genetics indicators in cancer and health status [21].

In addition, the scarcity of effective drugs and the refractory cancerous cells to the existing medicines challenge CRC treatment [22]. Immense studies demonstrated that complementary medicine may be a potential strategy for improving and managing CRC conditions [23]. Hence, physical activity and supplement substances with anticancer medications have been shown to augment therapeutic effectiveness and mitigate the systemic toxicity associated with chemotherapy medicines [24]. Several studies indicated that exercise training and physical activity can ameliorate the quality of life of patients with cancer and diminish the fatigue associated with cancer [25, 26]. While there are growing studies, the precise impact of heightened physical activity on cancer biology remains incomplete due to the challenges associated with extrapolating findings from mechanistic investigations to clinical studies [27].

Sparassis latifolia (SLPs) is classified as a scarce, therapeutic, consumable fungus characterized by a fruiting structure with a substantial nutritional composition encompassing numerous bioactive compounds [28]. Polysaccharides derived from SLPs are a type of edible fungus polysaccharides that are abundant in β-glucan. These polysaccharides have been identified as the primary nutrient component in SLPs [28]. Previous studies have demonstrated that SLPs exhibit anticancer effects in mice with vascular dilatation and bleeding reactions [29, 30]. Evidence has indicated that consuming the β-glucan derived from SLPs may alter cytokine levels in the spleen [31].

Therefore, based on the systems biology and artificial intelligence, we evaluated the protein–protein interaction (PPI) networks associated with colorectal cancer (CRC) susceptibility and occurrence. This work comprehensively investigates the role of microRNAs (miRNAs) and long non-coding RNAs (lncRNAs) as possible biomarkers and central nodes in CRC in both systems biology survey and experimental approaches. Furthermore, we have elucidated the significance of exercise training and Sparassis latifolia's bioactive compounds as supplementary approaches in improving immune function and hub genes associated with colorectal cancer (CRC) using pharmacophore modeling and molecular docking techniques.

## Material and methods

### Ethical code

This study conducted all procedures based on the Research Ethics Committees of the Islamic Azad University—Isfahan (Khorasgan) Branch (IR.IAU.KHUISF.REC.1402.182).

### Study plan

Data mining and bioinformatic analysis were conducted to detect the signaling pathway and hub genes involved in colorectal cancer and chemotherapy with 5-FU. In this phase, we constructed the protein–protein mapping and found the critical hub genes in colorectal cancer via system biology analysis. Moreover, the non-coding RNAs associated with inflammation, oxidative stress, extracellular structure organization, collagen formation, and assembly of collagen fibrils in colorectal cancer were explored. In the second stage, we conducted the in-vivo study and measured the expression pattern of hub genes and non-coding RNAs in the wet lab.

We provided 36 C57bl/6 male mice (6–7 weeks) from Royan Institute, Isfahan, IRAN. Mice were housed in the animal lab of the Islamic Azad University-Isfahan (Khorasgan) Branch. Mice were kept under standard conditions (12 h light–dark cycle, temperature of 24 ± 3 °C, and humidity of 65% ± 5). Furthermore, mice were received ad libitum to water and foods.

After one week of adaption, mice were divided into six groups (n = 6):

1. The Normal group was not treated. We called this group: (the control group).
2. The mice were induced colorectal cancer via one intraperitoneal (IP) administration of AOM (10 mg/kg b.w.). Subsequently, 3% DSS was consumed for one week in drinking water. We called this group: (the COL group).
3. COL mice were injected with chemotherapy (5-FU, 150 mg/kg, b.w.). We called this group: (the COL + Chem group).
4. Chemotherapy-treated COL mice were gavaged bioactive compounds of sparassis latifolia for eight weeks (400 mg/kg⋅bw⋅d). We called this group: (COL + Chem + BAC group)
5. Chemotherapy-treated COL mice were treated to exercise training with low to moderate intensity on the treadmill for eight weeks. We called this group: (COL + Chemo + EXr group).
6. Chemotherapy-treated COL mice were treated with sparassis latifolia bioactive compounds and exercise training for eight weeks. We called this group: (COL + Chem + BAC + EXr group).

At the end of the experiments, the mice were euthanasia under intraperitoneal administration of ketamine (100 mg/kg, body weight per mouse) and xylazine (10 mg/kg, body weight per mouse). It should be noted that tissues and blood were collected and stored at -80 °C for further experimental assay.

### System biology prediction

#### RNA-seq data analysis

The present study employed a methodology that involved utilizing network visualization techniques to analyze gene expression data, resulting in a model depicting the progression of colorectal cancer (COL). Biochemical compounds potentially influencing patients' life expectancy and survival rates were found. The investigation of pathological processes involved in the development of COL encompassed the examination of ceRNA interactions, the carcinogenic effects of AOM, and the underlying molecular signaling systems that play a crucial role in the pathogenesis of this disease. RNA-seq incorporates Next-Generation Sequencing (NGS) technology to determine mRNA expression. By transcribing complementary DNA from the mRNA generated through tissues and cells, the complementary DNA sequence may be monitored to identify the genes responsible for producing the mRNA [32]. The Cancer Genome Atlas (TCGA) is a large-scale project that archives patient data and tissue samples from various cancer types. The platform offers publicly accessible experimental data on copy number variations, DNA methylation, gene expression RNA-seq, and clinical aspects [32, 33].

Further, Microarray technology, established by Affymetrix and Illumina business enterprises, is required to analyze mRNA levels and gene expression profiles [34]. The approach employs many probes on a microarray platform to detect transcripts with considerable mRNA transcription, providing probe-sets with several customized probes for each gene [35]. Microarray data is commonly employed in assessments and is supported by strong statistical approaches and proven protocols [36]. The majority of microarray information derived from studies has been publicly available and archived in databases, especially Gene Express Omnibus (GEO) [37, 38].

The gene expression data and clinical data of colorectal adenocarcinoma (COAD) were obtained from The Cancer Genome Atlas (TCGA) using the TCGAbiolinks package using the R programming language software [39]. Overall, 483 samples of colorectal adenocarcinoma and 41 Normal tissue adjacent to a tumor have been acquired. Data normalization was conducted using the TMM method [40]. Genes with insignificant expression levels were removed using the edgeR package, following the CPM criterion (Count/million) [41]. Finally, the data involving the expression of each gene in the samples were transformed into a logarithmic form based on 2 applying the limma package and archived as the normalized expression matrix.

The samples in the colorectal adenocarcinoma were grouped into normal and malignant groups based on specific clinical characteristics such as sample barcoding and M pathological indexes. The linear model approach was applied to evaluate the differential expression between malignant and normal samples for all variables. Genes suggesting significant expression differences between cancer and normal samples were selected according to a threshold of adj P.value < 0.01 and logFC ± 1.

#### Microarray data analysis

The microarray profiles related to colorectal cancer were obtained from the GEO database, accessible at https://www.ncbi.nlm.nih.gov/geo, using the search term "Colorectal Cancer". The analysis of samples from GSE110224 [42] was conducted using the R programming language software and Bioconductor packages. These datasets consisted of 3 primary adenocarcinomas and three samples matched normal samples from each patient. This analysis aimed to identify genes that were differentially expressed (DEGs) in these samples. The employment of the MAS5 approach played a crucial role in normalizing the data [43]. The datasets underwent a comparative analysis using a t-test to identify genes with substantial differential expression. A significance threshold of *P* < *0.05* was applied. The ggplot tool generated a heatmap diagram, highlighting genes with a *P-value* < *0.001*. In contrast, we employed a log fold change (logFC ± 2.0) cut-off to cluster the overexpressed and downregulated genes, thereby identifying the genes of considerable importance.

In the subsequent phase, we utilized the STRING 11.5 database to construct a network of protein–protein interactions (PPIs) comprising hub nodes derived from each dataset study, focusing on medium confidence 0.4 as outlined by Szklarczyk et al. [44]. The identification of hub genes based on betweenness centrality:0.006, degree:5, and closeness centrality:0.2 was performed using CytoScape 3.6.0 to visualize the network parameters [45]. Cytoscape is an open-source computational biology platform aimed at visualizing biological interaction networks based on network parameters and combining them with gene expression profiles and other statistics. Supplementary functionalities may be accessed using plugins [46].

The design of the genetics network of hub nodes was informed by network diameters, eigenvector centrality, and modularity class, with the aim of effectively visualizing and manipulating huge graphs using the Gephi software 9.2.0 platform [47]. Gephi is a highly capable software tool designed to view and analyze networks [48]. The gene set enrichment analysis in the Enrich-KG database was used to identify the important molecular signaling pathways and gene ontology processes related to hub genes that exhibited significant differential expression in colorectal cancer [49]. Subsequently, the genetic network, including these hub genes, was constructed using Gephi software 9.2.0.

#### Generation of a gene expression panel associated with Colorectal carcinoma risk

Gene overlaps in the microarray data and RNA-seq based on significant cut-off were analyzed using a Venn diagram [50]. Each protein–protein interaction network in STRING is marked with one or more 'scores' [44]. These ratings do not reflect the degree or specificity of the interaction. The indicators are measures of confidence, namely how probable STRING thinks an interaction to be true based on the facts and evidence. All ratings range from 0 to 1, with 1 being the maximum level of confidence [44]. A score of 0.5 suggests that about every other interaction might be incorrect, meaning it could be a false positive [44]. The genetic interconnections network was established with the STRING 11.5 server based on a medium confidence score (0.4) and analyzed in the CytoScape application using network parameters (Degree: 20, Betweenness Centrality: 0.005, and Closeness Centrality: 0.2) [51]. Gephi applications finalized the network, and potential indicators for molecular screening and pharmacological targets for drug design were discovered [47]. The cellular, molecular, and signaling pathways corresponding to the switchable genes were analyzed by employing data enrichment in Enrich-R and KOBAS servers. The Gephia server was applied to explore the correlation between the expression of candidate genes (FN1, CXCL8, IL1β, and ITIH4) and the with mortality rate based on overall survival score [52]. This study examined the significance of FN1, CXCL8, IL1β, and ITIH4 in conducting a comprehensive analysis of tumor-infiltrating immune cells using the Tumor IMmune Estimation Resource (TIMER) Web Server [53].

#### LncRNA prediction

In this study, we comprehensively examined several databases, including LncRNADisease [54], LncTard [55], lncHUB2 [56], MNDR [57], LncSEA [58], RNAInteractome [59], and LNCBOOK [60]. Our objective was to identify potential associations between long non-coding RNAs (lncRNAs), hub genes, and colorectal cancer, specifically focusing on their role as post-transcriptional regulatory agents. In order to construct a putative competing endogenous RNA (ceRNA) network involved in the etiology of colorectal cancer, the miRNet database [61] was utilized to identify commonly expressed genes and anticipated lncRNAs.

#### ceRNA network

miRNet 2.0 is a user-friendly online application available at https://www.mirnet.ca. It enables users to effortlessly generate and graphically analyze miRNA-related regulatory networks [62]. The software was first launched in 2016 to analyze miRNA-target gene networks using both computational predictions and strong experimental validations. It has been regularly updated in response to the increasing demands of the community. The 2.0 edition includes 13 modules categorized into 4 groups based on input types [62]. In conclusion, we identified the most significant lncRNA and microRNA inside the network by considering its degree and functional predictions. These predictions were derived from analyzing lncRNA-gene co-expression correlations using the lncHUB server [56].

The analysis of GEO and TCGA data was assessed by employing the R programming language to establish expression differences across groups and assess the significance of the one-way ANOVA test. A t-test was employed to analyze the disparity in gene expression between cancer groups and the normal group. The association between gene expression and the patient prognosis was analyzed using the Kaplan–Meier graph and logRank test [63].

### Pharmacophore modeling and molecular docking

The identification of draggability interactions with genes/proteins was accomplished by the utilization of the Drug Genes Interaction database (DGIdb) available at https://www.dgidb.org [64]. In contrast, the authors of the study conducted by Gilson et al. (2016) successfully compiled a comprehensive inventory of chemical compounds that specifically interact with druggable cut-points within the binding database [65]. This compilation was based on the evaluation of IC50 values and protein classifications. Based on the aforementioned knowledge, it is now possible to strategically identify and target druggable genes within the pathomechanism network using homologous natural antagonists, which could potentially lead to enhanced survival rates. In this study, we conducted an analysis to discover targets that have a negative linear correlation with survival rates, intending to identify potential pharmaceutical synergistic effects. The genes that exhibited the highest betweenness centrality were identified as potential targets for drug development and the selection of antagonists based on drug design principles. The protein data bank (PDB) [66] repository was utilized to access and examine the X-ray diffraction structure of the protein FN1 (PDB ID: 3M7P), which was experimentally determined and represented in three-dimensional form. The most bioactive components were discovered based on the literature review. The procedure for extracting and storing active chemicals in a three-dimensional structural format (SDF) from the PubChem server [67] and depositing them in the chemical library was conducted utilizing the Open Babel [68]. Prior to determining the binding affinity between the macromolecule FN1 and the chemical library, we conducted the necessary steps to prepare and optimize the 3D structure of FN1. This involved eliminating additional chains, non-complex compounds, ligands, ions, and solvents using the Dock Prep tools available in UCSF Chimera 1.8.1 [69]. Subsequently, following the process of energy minimization and translation of the chemical library from SDF format to PDB-QT format, the molecular docking estimation was conducted using the PyRx virtual screening platform [70]. The criteria for selecting the optimal therapeutic target were a binding affinity of less than negative five and a root mean square deviation (RMSD) of less than 2, as determined using molecular docking analysis. For detection interaction between medicinal chemistry compounds of phytochemicals cocktail and macromolecules (FN1), we applied PyMOL [71] and BIOVIA Discovery Studio Visualizer software version 2021.

In contrast, we employed the Schrödinger-Maestro 11.5 software tools [72] to generate appropriate pharmacophore models for the FN1 protein. The pharmacophore models were constructed using Ligprep, Phase Pharma, the OPLS 2005 force field, active ligands with an IC50 threshold of less than 1000 as lead ligands, and a range of 4 to 5 features. The pharmaceutical screening process also demonstrated the correspondence between bioactive molecules and the pharmacophore models.

### Mouse model for colorectal carcinoma

The dosage of Azoxymethane (AOM) (No. 684–93-5, Sigma Aldrich, USA) was chosen as 10 mg/kg b.w. in accordance with the previous studies [73].

The mice were administered one intraperitoneal (IP.) AOM. Subsequently, 3% DSS was received for 7 days, and then returned to normal drinking [73].

### Drug treatment using 5-FU

A single dose of 5-Fluorouracil (5-FU) solution (No. 33069–62-4, Sigma Aldrich, USA) was injected (50 mg/kg) intraperitoneally [74]. Following the induction of the colorectal cancer model in mice, the respective groups were administered 5-FU for a duration of one day.

### Evaluation of biomarker quantitation (ELISA assay)

Blood samples were taken from the right ventricle of each mouse. Serum was extracted by centrifuging blood samples at 1600 g for 15 min at 4 °C in tubes. The concentrations of IL-18 (ab216165, Abcam), IL-13 (ab219634, Abcam), IL-17 (ab100702, Abcam), IL-2 (ab100706, Abcam), GPX (MBS456700, MyBioSource) and SOD (ab285309, Abcam) were determined using enzyme-linked immunosorbent assay as per the manufacturers' recommendations.

### Extraction protocol of sparassis latifolia

The isolation of the polysaccharides was conducted by other studies [75]. The extraction of the residues was conducted using a 500 mL solution of sodium hydroxide (NaOH) with a concentration of 1 mol/L. The extraction process was performed at a temperature of 60 ◦C and lasted for 30 min, during which the mixture was adequately stirred. Following the filtration process, the remaining substance underwent two further washes with distilled water. The resulting liquid was then subjected to neutralization using hydrochloric acid (HCl) at a concentration of 6 mol/L. Subsequently, the liquid portion of the mixture was subjected to a process of concentration, dialysis, and freeze-drying to acquire the NaOH extract, referred to as NSP. The filtrate layer underwent an additional extraction process using 500 mL of hydrochloric acid with a concentration of 0.5 mol/L [75]. This extraction was carried out at a temperature of 100 ◦C for 1 h in a water bath. Subsequently, the resulting suspension was filtered and subjected to another round of washing using distilled water. The supernatant that was gathered underwent concentration and dialysis procedures after neutralization with a 1 mol/L solution of sodium hydroxide (NaOH). Following this, freeze-drying was performed to acquire the HCl extract, which was assigned the code HSP. Subsequently, the remaining substances were separated by using an additional 500 mL of potassium hydroxide (KOH) solution with a concentration of 1 mol/L. This extraction process was conducted for 30 min at a temperature of 60 ◦C. The resulting mixture was then subjected to a series of purification steps, including washing with distilled water, followed by centrifugation, concentration, and dialysis. The resulting fraction produced from this process was designated as KSP [75].

### Evaluation of pathological phenomena

After the mice were sacrificed, their gastrointestinal tissues were promptly preserved in 10% buffered formalin solution and then embedded in paraffin. In addition, the tissues were subjected to fixation and then sectioned into sections with a thickness of 5 µm. Following the process of deparaffinization and hydration, the tissue slices were subjected to staining using H&E. Subsequently, the stained sections were examined using light microscopy.

### qPCR real-time assay

The extraction of total RNAs from stomach tissue was performed using a TRIzol reagent (Thermo Scientific, USA). To mitigate the presence of genomic DNA contamination, the samples were subjected to treatment with TaKaRa DNaseI, a product manufactured by TaKaRa from Japan. The cDNA synthesis kit manufactured by TaKaRa was used to produce complementary DNA (cDNA) from 1 μg of total RNA via the process of reverse transcription of messenger RNA (mRNA), following the directions provided by the manufacturer. The q-RT PCR method was performed using an Applied Biosystems real-time PCR thermal cycler (Thermo Fisher Scientific, Waltham, MA, USA) and SYBR green dye (TaKaRa, Japan). The 2^−ΔΔct^ technique was used to assess gene expression. The 18 s rRNA and U6 was used as a housekeeping gene, serving as an internal control, for the purpose of determining relative quantification, as specified. The primers were procured from Micro-gene, a Korean business. Table 1 displays the sequences associated with the set of primers.

**Table 1:** The sequence of primers used in the study

Gene	Sequence of Primers	Annealing temperature
GAPDH	Forward: 5ʹ-TGCCGCCTGGAGAAACC-3'	60 °C
	Reverse: 5ʹ-TGAAGTCGCAGGAGACAACC-3'	
U6	Forward: 5ʹ-GCTTCGGCAGCACATATACTA-3'	60 °C
	Reverse: 5ʹ-CGAATTTGCGTGTCATCCTTG-3'	
SNHG16 (NR_027821)	Forward: 5ʹ-TCCTCCTCCTTGGGTGCTCT-3'	60 °C
	Reverse: 5ʹ-CCTTACATCCCTGCCTCCTCTA-3'	
KCNQ1OT1 (NR_001461.5)	Forward: 5ʹ-GCACTCTGGGTCCTGTTCTC-3'	60 °C
	Reverse: 5ʹ-CACTTCCCTGCCTCCTACAC-3'	
miR-132-3p (MI0000158)	Forward: 5ʹ-CAGTCTACAGCCATGGTC-3'	60 °C
	Reverse: 5ʹ-GAACATGTCTGCGTATCTC-3'	
PVT1 (NR_003368.2)	Forward: 5ʹ-ATCCACCCTCTTGTCTGATTTTCT-3'	60 °C
	Reverse: 5ʹ-AATCCACAGGGTTCAGGAAGTC-3’	60 °C
Col1a2 (NM_007743)	Forward: 5ʹ-TTCTGTGGGTCCTGCTGGGAAA -3’	60 °C
	Reverse: 5ʹ-TTGTCACCTCGGATGCCTTGAG -3'	
Itih4 (NM_018746)	Forward: 5ʹ-CCTTTCCTGGAGAAGATGGCAC-3’	60 °C
	Reverse: 5ʹ-CTGATGAGAGCAGTGGATTGGC -3’	
Mmp7 (NM_010810)	Forward: 5ʹ-AGGTGTGGAGTGCCAGATGTTG -3’	60 °C
	Reverse: 5ʹ-CAGTGAAATTCTTGACCGCTTTCC-3'	
Msx2 (NM_013601)	Forward: 5ʹ-AAGACGGAGCACCGTGGATACA -3’	60 °C
	Reverse: 5ʹ-CGGTTGGTCTTGTGTTTCCTCAG-3’	
NEAT1	Forward: 5ʹ-GCTCTGGGACCTTCGTGACTCT-3'	60 °C
	Reverse: 5ʹ-CTGCCTTGGCTTGGAAATGTAA-3’	

### Statistical analysis

The data provided in this study were represented by the mean and standard deviation. The statistical analysis was conducted with GraphPad Prism (Version 9). Normalizing the distribution was conducted utilizing the Shapiro–Wilk test, which revealed that the variables exhibited a normal distribution. The data underwent a one-way analysis of variance (ANOVA), and Tukey's post hoc test was employed to account for multiple comparisons. In addition, the data underwent evaluation using Pearson correlation to analyze the relationship between mRNAs and non-coding RNAs. In this study, the linear regression analysis method was employed to examine the relationship between the expression level of the Fn1 and potential biomarkers. The linear regression model was specified to provide the model equation, and estimation was carried out using the least squares method. The statistical analysis used a t-test and F-test to assess the performance of the linear regression model.

It should be noted that the t-test was used to test the significance of regression coefficients in the model, and the F-test was used to test the significance of the entire regression model. Moreover, the R2 was used to estimate the degree between the fitted curve and the original data. Statistical significance was attributed to the results when the p-values were < 0.05.

## Result

### Conclusions from a system biology approach

**Fig. 1:**
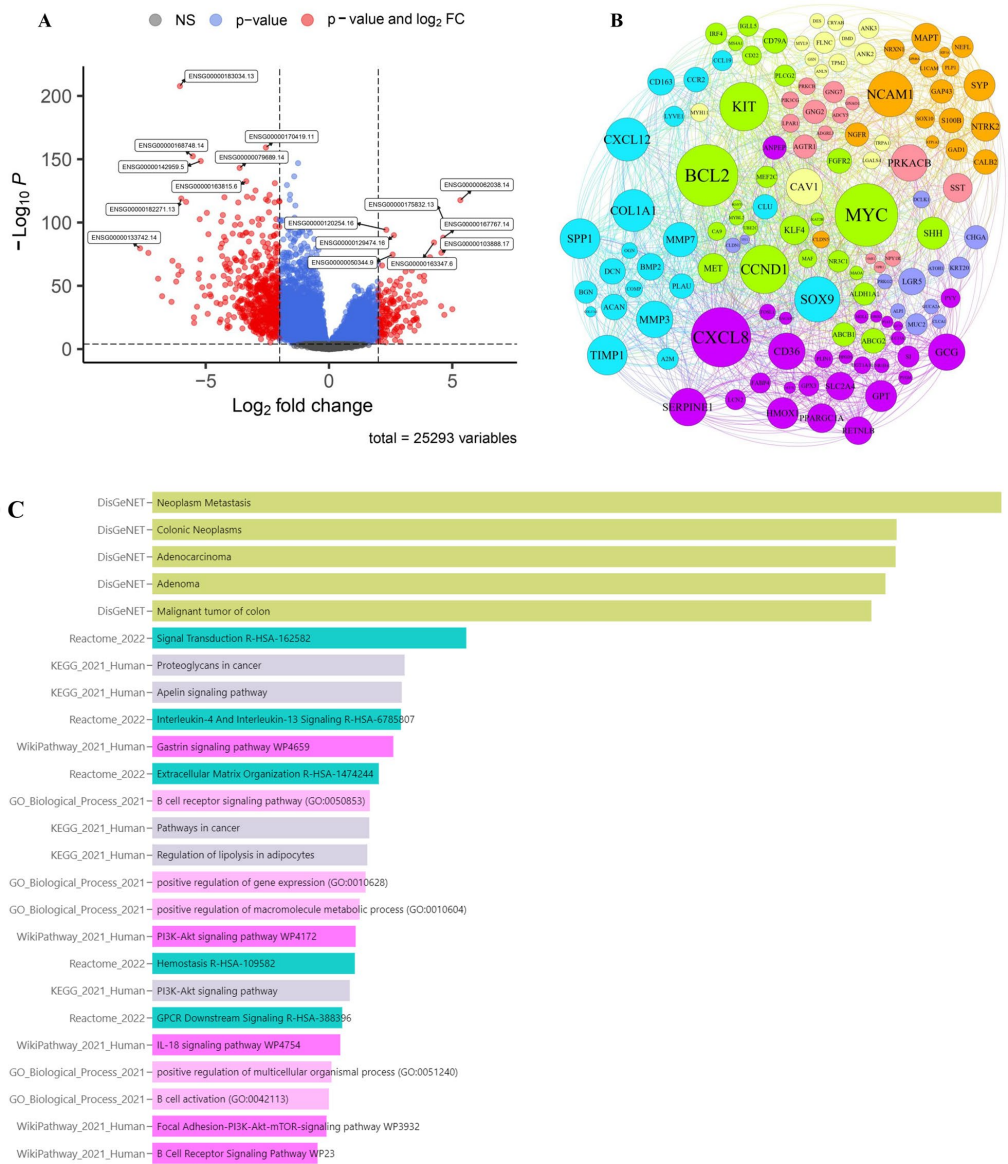
TCGA-COAD analysis and enrichment assessment.** A** The visualization of significant DEG in colorectal cancer based on RNA-seq is depicted in the volcano plot, with consideration of a logFC ± 2. Volcano plots depicting differential expression of all transcripts in colorectal cancer (CRC) relative to normal tissue are discussed using The Cancer Genome Atlas (TCGA) data. Genes received acceptance based on criteria of logFC ± 1 and adj P.value < 0.01 to identify genes potentially involved in tumorigenesis processes. Variations in gene expression patterns have been correlated to the prognosis of individuals with CRC. **B** The protein–protein interactions (PPIs) network of 133 hub genes in colorectal cancer development and progression (TCGA-COAD) was presented based on network diameters (Degree = 10, betweenness centrality = 0.005, and closeness centrality = 0.2). **C** The enrichment analysis showed a higher abundance of hub genes related to adenoma/adenocarcinoma, malignancy, metastasis, extracellular structure organization, PI3K-Akt signaling pathway, IL-18, cancer pathway, GPCR downstream signaling, and B-cell activation

Our team focused on the possible impact of differentially expressed genes on the recurrence of colorectal cancer. The TCGA COAD dataset was used to identify important differentially expressed genes using the limma package analysis. We identified 15,096 genes with a significant score adjP.value < 0.01 in the bioinformatics examination. We identified 288 genes with overexpression patterns and 955 genes with downregulation patterns based on the log FC ± 1.0 cut-off. The volcano plot displays the distribution of substantially differentially expressed genes after normalization and statistical analysis using a logFC ± 2 threshold (Fig. 1A). After creating the protein–protein interaction (PPI) network using the STRING web server and analyzing the metrics (Degree = 10, betweenness centrality = 0.005, and closeness centrality = 0.2) with the CytoScape tool, 133 hub genes were discovered. Figure 1B displayed the hub genes based on network properties, namely eigenvector and modularity class. The enrichment analysis showed a higher abundance of hub genes related to adenoma/adenocarcinoma, malignancy, metastasis, extracellular structure organization, PI3K-Akt signaling pathway, IL-18, cancer pathway, GPCR downstream signaling, and B-cell activation (Fig. 1C).

**Fig. 2:**
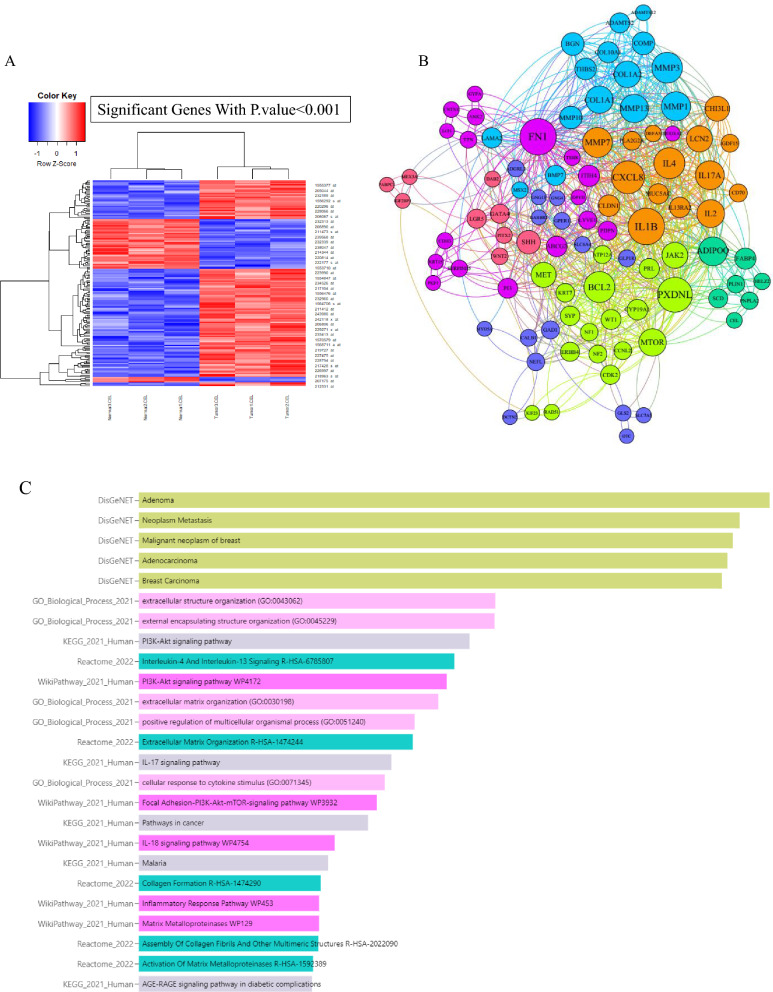
Data set analysis and enrichment assessment.** A** The visualization of significant DEG in colorectal cancer based on the microarray dataset is depicted in the heatmap diagram, with consideration of a P-value < 0.001. **B** The genetic network of 97 hub genes in colorectal cancer development and progression (GSE110224) was presented based on network diameters (Degree = 5, betweenness centrality = 0.006, and closeness centrality = 0.2). **C** The verification of hub gene's function in biological systems confirmed their involvement in a range of processes, including adenoma/adenocarcinoma, malignancy, metastasis, extracellular structure organization, PI3K-Akt signaling pathway, IL-4/IL-13/IL-17/IL-18, inflammation and inflammatory response pathway, matrix metalloproteinases, and AGE-RAGE signaling pathway

In order to investigate the potential adverse effects of exposure to AOM/DSS as a carcinogenic substance on colon tissue, an analysis was conducted on the dataset GSE110224. In this particular scenario, we have successfully identified and documented a total of 2870 genes that exhibit substantial differential expression in the primary tumor samples, as compared to the adjacent normal samples. Based on the log FC ± 2.0 cut-off, we identified a total of 599 genes exhibiting overexpression patterns and 164 genes displaying downregulation. The heatmap in Fig. 2A represents the distribution of significantly differentially expressed genes after normalization and statistical analysis, with a threshold of P.value < 0.001. Upon constructing the protein–protein interaction (PPI) network using the STRING web server and then displaying the metrics (Degree = 5, betweenness centrality = 0.006, and closeness centrality = 0.2) in the CytoScape program, a total of 97 hub genes were identified. The hub genes were shown in Fig. 2B using network characteristics, namely eigenvector and modularity class. The enrichment analysis revealed an increased presence of hub genes associated with adenoma/adenocarcinoma, malignancy, metastasis, extracellular structure organization, PI3K-Akt signaling pathway, IL-4/IL-13/IL-17 /IL-18, inflammation and inflammatory response pathway, matrix matalloproteinase, and AGE-RAGE signaling pathway (Fig. 2C).

**Fig. 3:**
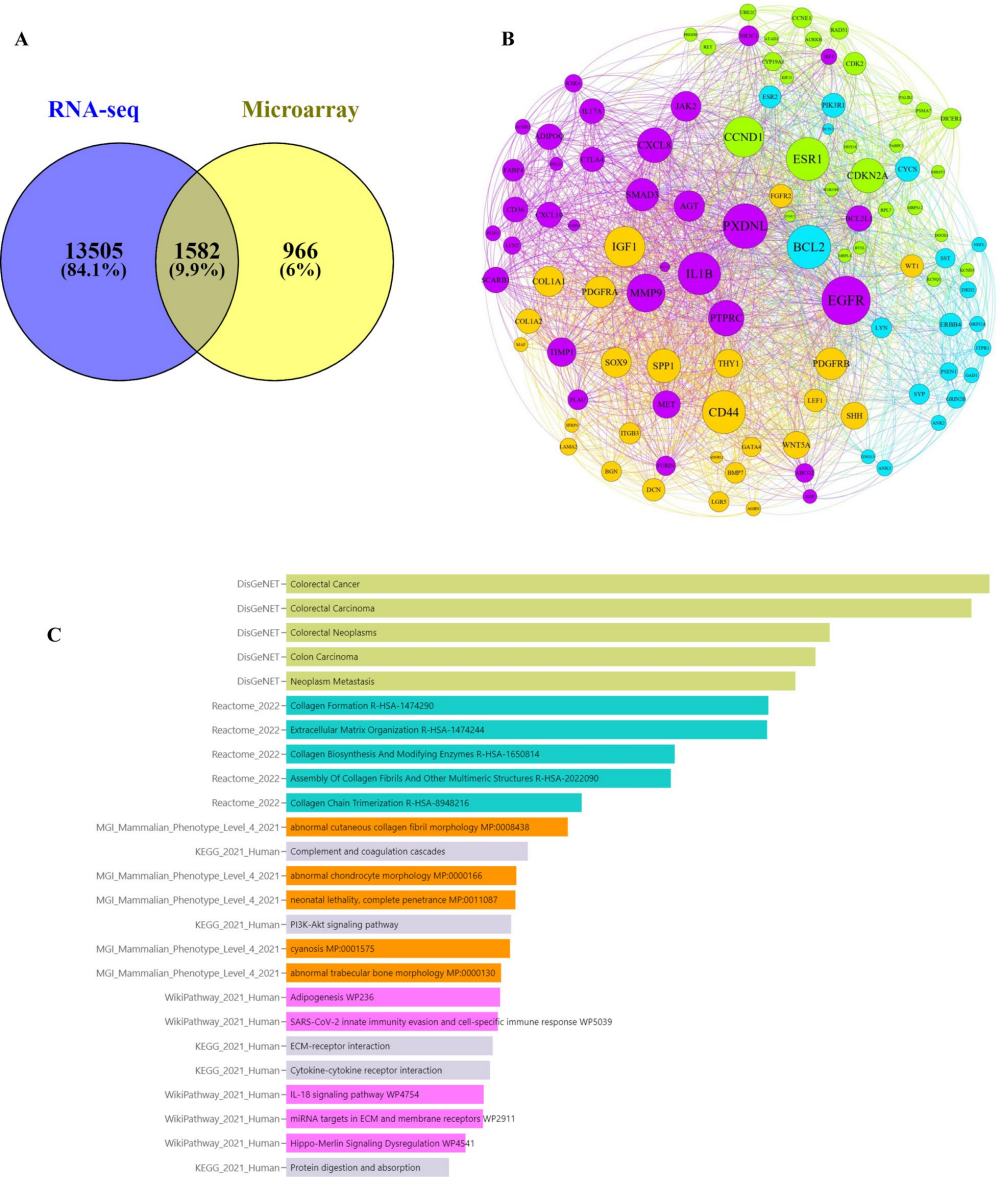
Generation of a gene expression panel associated with Colorectal carcinoma risk based on common genes between RNA-seq and microarray data.** A** The VENN diagram bioinformatics tool was used to discover common genes that were rough across RNA-seq and Microarray datasets, revealing 1582 common genes from significant gene lists based on adj p-values. **B** The 104 hub genes interactions network is generated in Gephi software 0.9.2 based on network parameters (Degree: 20, Betweenness Centrality: 0.005, and Closeness Centrality: 0.2), eigenvector, and modularity class. Through bioinformatics research, we have found important biomarkers in this network that might serve as valuable indicators for prognosis, diagnosis, and monitoring. **C** According to the enrichment facts, 104 hub genes correspond with colorectal cancer, cancer metastasis, collagen formation, extracellular matrix organization, PI3K/Akt signaling pathway, ECM receptors, Cytokine-cytokine receptor interaction, IL18 signaling pathway, and Hippo signaling pathway

The VENN diagram bioinformatics tool was used to discover common genes that were rough across RNA-seq and Microarray datasets, revealing 1582 common genes from significant gene lists based on adj p-values (Fig. 3A). The 104 hub genes interactions network is generated in Gephi software 0.9.2 based on network parameters, eigenvector, and modularity class (Fig. 3B). Through bioinformatics research, we have found essential biomarkers in this network that might serve as valuable indicators for prognosis, diagnosis, and monitoring. Furthermore, according to the enrichment facts, 104 hub genes correspond with colorectal cancer, cancer metastasis, collagen formation, extracellular matrix organization, PI3K/Akt signaling pathway, ECM receptors, Cytokine-cytokine receptor interaction, IL18 signaling pathway, and Hippo signaling pathway (Fig. 3C).

**Fig. 4:**
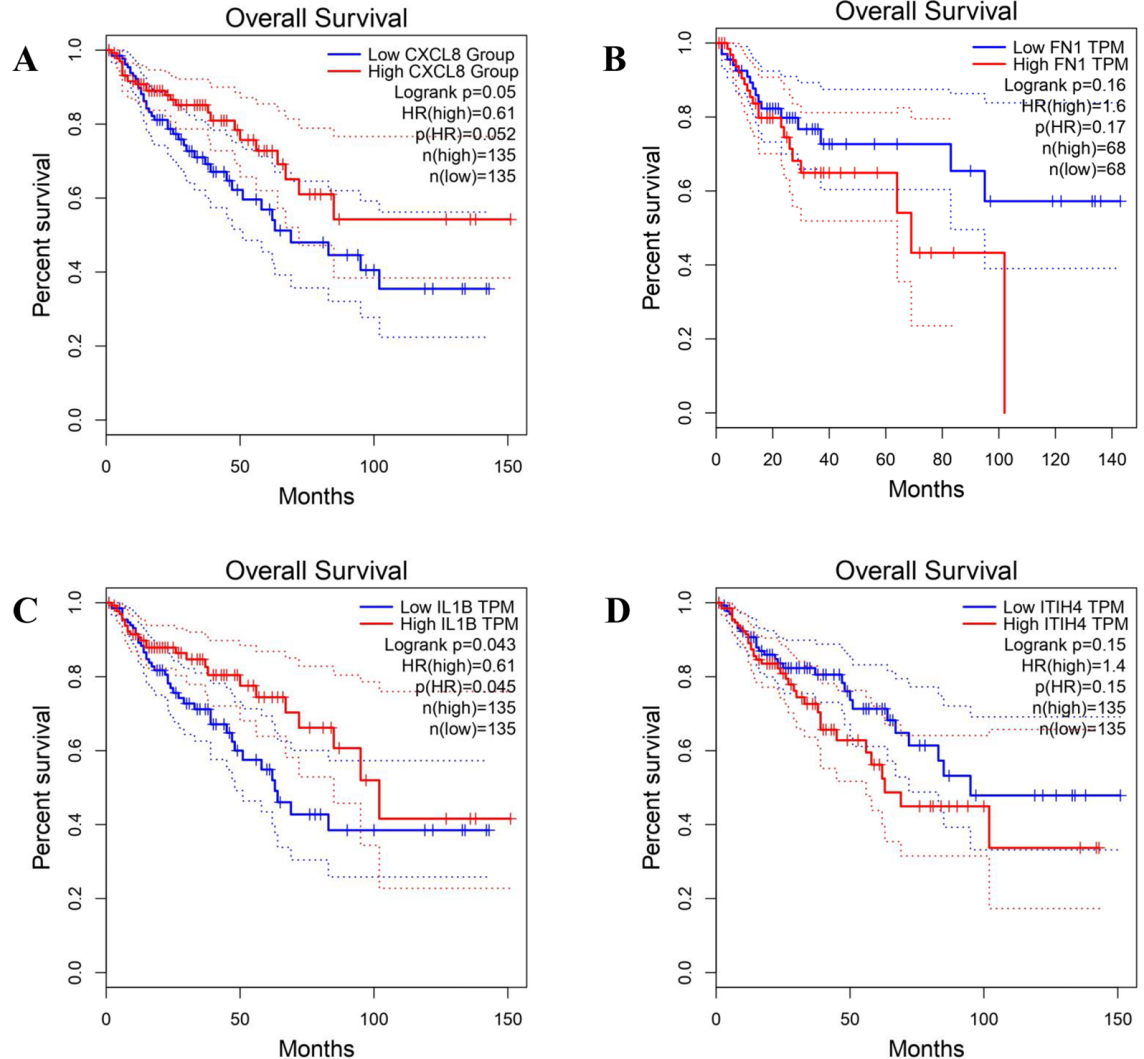
Predicting the Survival Rate of Patients with CRC based on the Level of Hub Genes.** A-D** The association between gene expression and the patient prognosis was analyzed using the Kaplan–Meier graph and logRank test. The Kaplan–Meier diagram is illustrated for high-risk patients & low-risk patients. The expression mortality risk was computed based on IL1β, CXCL8, FN1, and ITIH4 expression, and the risk score median was applied as a cut-off value. The four-hub gene panel combination exhibited a stronger predictive value, with patients with lower levels of expression at CXCL8 and IL1β having a poorer overall survival (OS) compared to overproduced those (log-rank test P-value:0.05 and 0.04). Moreover, patients with overexpression expression at FN1 and ITIH4 have poorer overall survival (OS) compared to lower levels of expression (log-rank test P-value:0.1)

The four-hub gene panel combination exhibited a more robust predictive value, with patients with lower levels of expression at CXCL8 and IL1β having a poorer overall survival (OS) compared to overproduced of those (log-rank test P-value:0.05 and 0.04, Fig. 4A and 4C). Moreover, patients with overexpression expression at FN1 and ITIH4 had a poorer overall survival (OS) compared to those with lower levels of expression (log-rank test P-value:0.1, Fig. 4b and d).

**Fig. 5:**
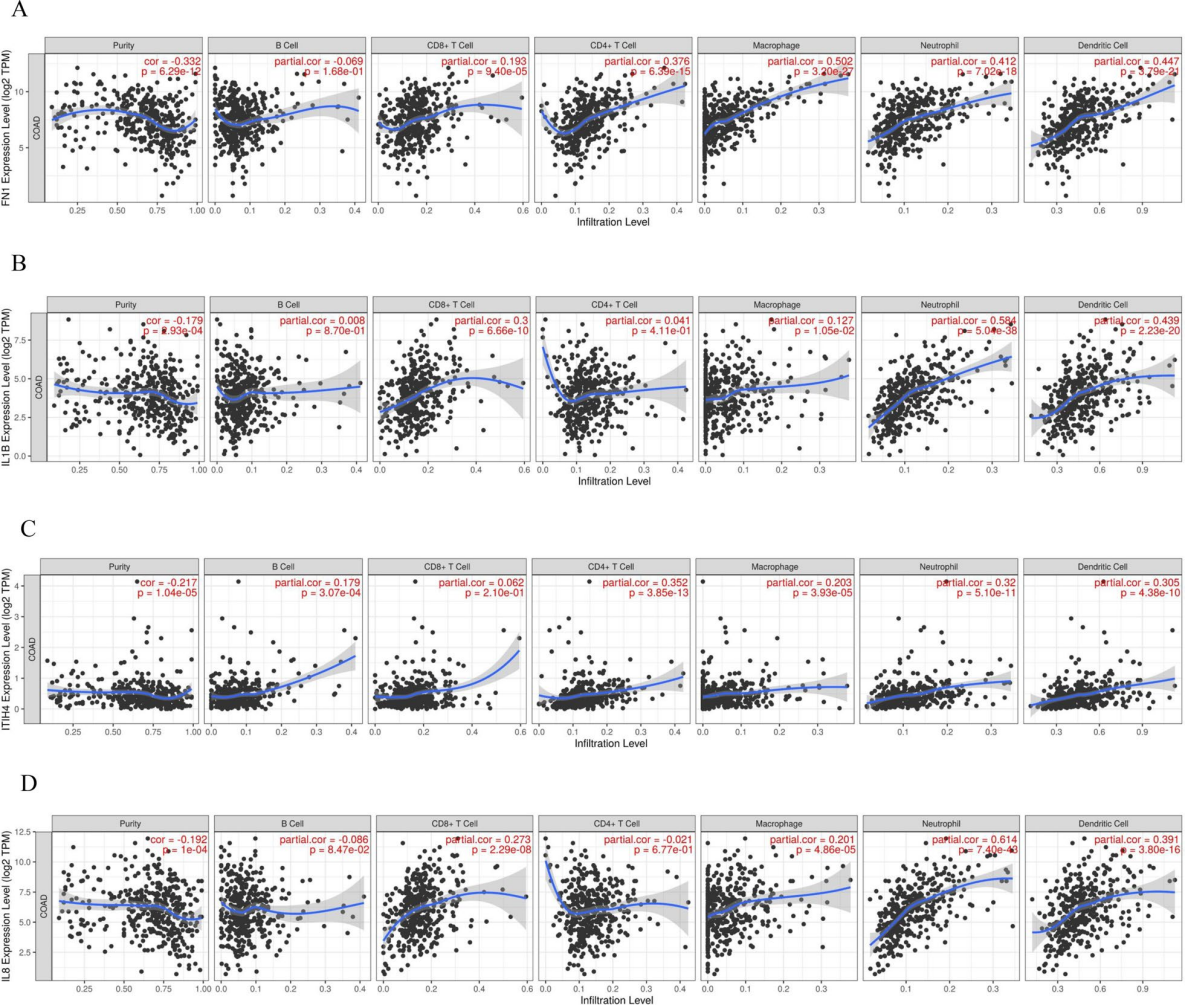
Comprehensive Analysis of tumor-infiltrating Immune Cells.** A-D** The correlation between Tumor-Infiltrating Immune Cells and FN1, IL1B, ITIH4, and CXCL8 is positive, as per our data—gene module to explore the correlation between gene expression and abundance of immune infiltrates in the online platform

Therefore, we used the tumor IMmune Estimation Resource (TIMER) Web Server to investigate FN1, CXCL8, IL1β, and ITIH4's function in the Comprehensive Analysis of tumor-infiltrating Immune Cells. Our results showed a favorable association between tumor-infiltrating immune cells and FN1, CXCL8, IL1β, and ITIH4 (Fig. 5A–D).

### The discovery of long non-coding RNAs and the screening of interactions in the competing endogenous RNA network

**Fig. 6:**
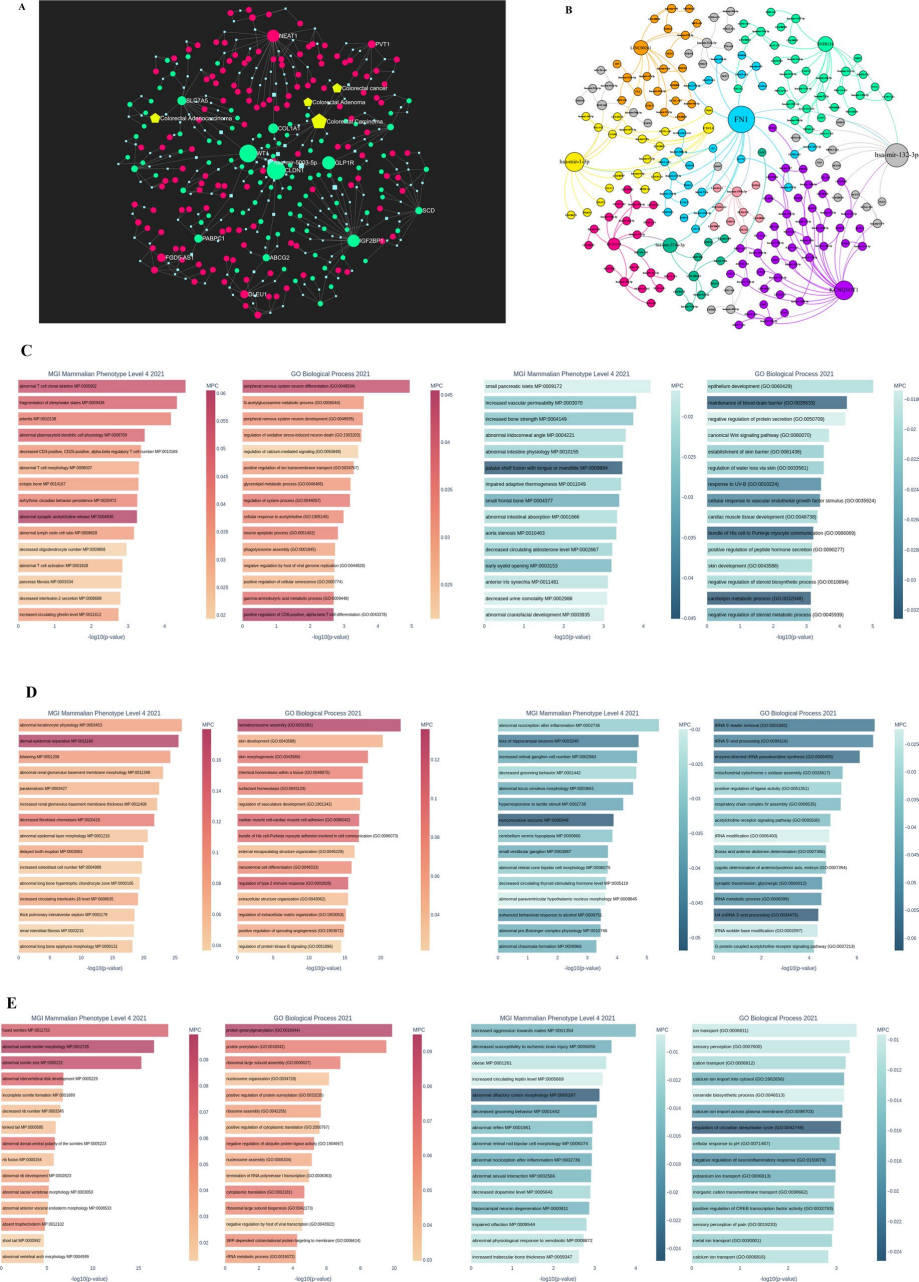

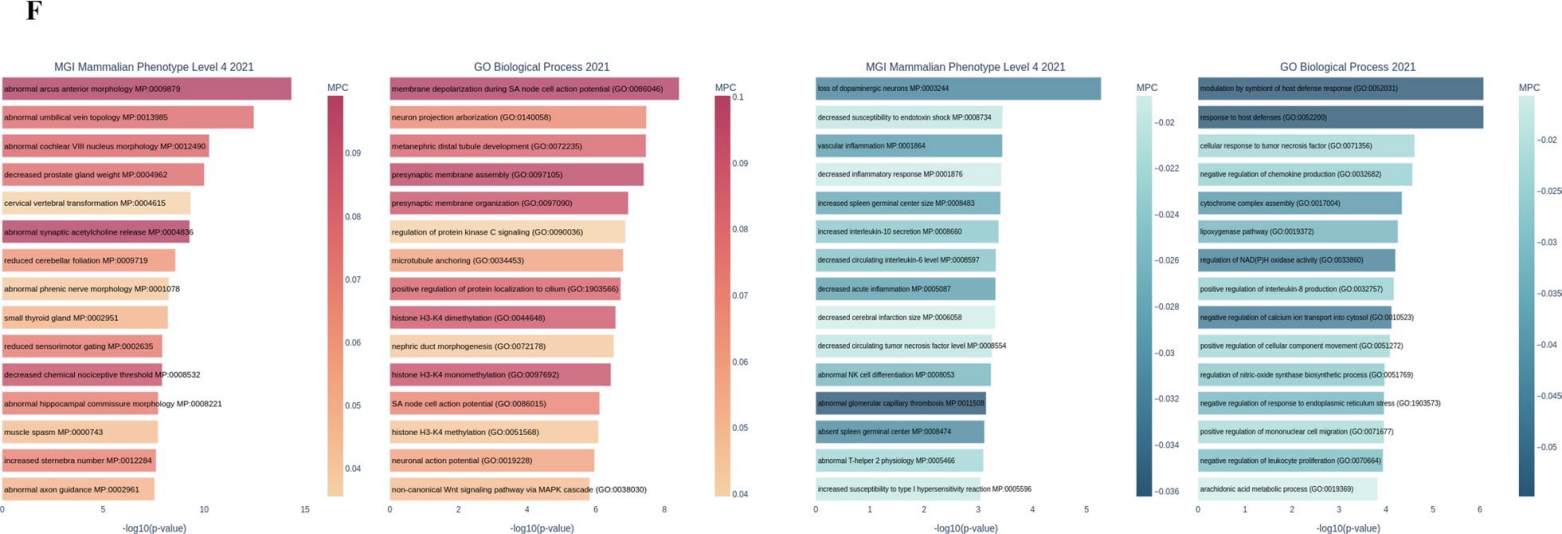
A potential ceRNA network between common hub genes and predicted lncRNA and microRNAs. **A**, **B** The ceRNA network emphasized significant lncRNAs and microRNAs in the pathogenesis of colorectal cancer. Our findings suggest that the lncRNAs PVT1, NEAT1, SNHG16, and KCNQ1OT1 target hub genes through the ceRNAs network based on the miRNet online platform. Moreover, the ceRNA network predicts the significant role of microRNA 132-3p in regulating this network. **C–F** The enrichment analysis of PVT1, NEAT1, SNHG16, and KCNQ1OT1 revealed that these lncRNAs serve as regulatory factors and are involved in the positive regulation of CD8^+^ and T-cell differentiation, positive regulation of cellular senescence, abnormal immune cells, and morphology, decrease IL-2 secretion, regulation of oxidative stress, regulation of apoptosis cell death, WNT signaling pathway, abnormal intestinal absorption, circulating IL-18 concentration, as well as several biological processes such as epithelial formation, cell adhesion, morphology, cell differentiation, extracellular matrix structure organization, and immune system response, positive regulation of translation process and negative regulation of ubiquitination complex activity, obesity, ion transport, negative regulation of neuroinflammatory response, positive regulation of CREB transcription factor activity, regulation of protein kinase C sigaling pathway, histone H3-H4 monomethylation/dimethylation, non cononical WNT signaling pathway via MAPK cascade, decrease susceptibility to endotoxone shock, decrease inflammatory response, increase IL-10 secretion, decrease circulating IL-6 level and TNF-α, decrease acute inflammation, abnormal NK-cell differentiation, and negative regulation of chemokine production

Long non-coding RNAs (lncRNAs) have been recognized as significant factors in cancer progression, as they play a role in regulating carcinogenesis, metastasis, and treatment resistance. Moreover, these lncRNAs often exhibit aberrant expression patterns due to a variety of molecular pathways. Furthermore, the varied expression patterns of long non-coding RNAs (lncRNAs) as modulators of posttranslational modifications may have significant implications in the development and treatment of cancer. Hence, using the pathomechanism network of colorectal cancer, we made predictions about a collection of long non-coding RNAs (lncRNAs) associated with diseases and target genes. Figure 6A and B illustrated the construction of a hypothetical competing endogenous RNA (ceRNA) network, including commonly expressed genes and anticipated long non-coding RNAs (lncRNAs), which serves to emphasize the importance of certain lncRNAs in the development of colorectal cancer pathogenesis. Our study revealed that long non-coding RNA PVT1, long non-coding RNA SNHG16, long non-coding RNA KCNQ1OT1, and long non-coding RNA NEAT1 have targeting capabilities towards hub genes via the competing endogenous RNA (ceRNA) network. The enrichment analysis conducted on PVT1, SNHG16, KCNQ1OT1, and NEAT1 in lncHUB revealed that the long non-coding RNA PVT1 has a role as a regulatory factor in the positive regulation of CD8^+^ and T-cell differentiation, positive regulation of cellular senescence, abnormal immune cells and morphology, decrease IL-2 secretion, regulation of oxidative stress, regulation of apoptosis cell death, WNT signaling pathway, and abnormal intestinal absorption (Fig. 6C). Furthermore, it has been shown that NEAT1 is linked to elevated levels of circulating IL-18 concentration, as well as several biological processes such as epithelial formation, morphology, cell adhesion, extracellular matrix structure organization, cell differentiation, and immune system response (Fig. 6D). SNHG16 has a role as a regulatory factor in the positive regulation of the translation process and negative regulation of ubiquitination complex activity, obesity, ion transport, negative regulation of neuroinflammatory response, and positive regulation of CREB transcription factor activity, as seen in Fig. 6E. Furthermore, it has been shown that KCNQ1OT1 is linked to the regulation of protein kinase C signaling pathway, histone H3-H4 monomethylation/dimethylation, non-cononical WNT signaling pathway via MAPK cascade, decrease susceptibility to endotoxin shock, decrease inflammatory response, increase IL-10 secretion, decrease circulating IL-6 level and TNF-α, decrease acute inflammation, abnormal NK-cell differentiation, and negative regulation of chemokine production. These findings are supported by the data presented in Fig. 6F.

### In silico pharmacophore modeling and drug discovery

**Fig. 7:**
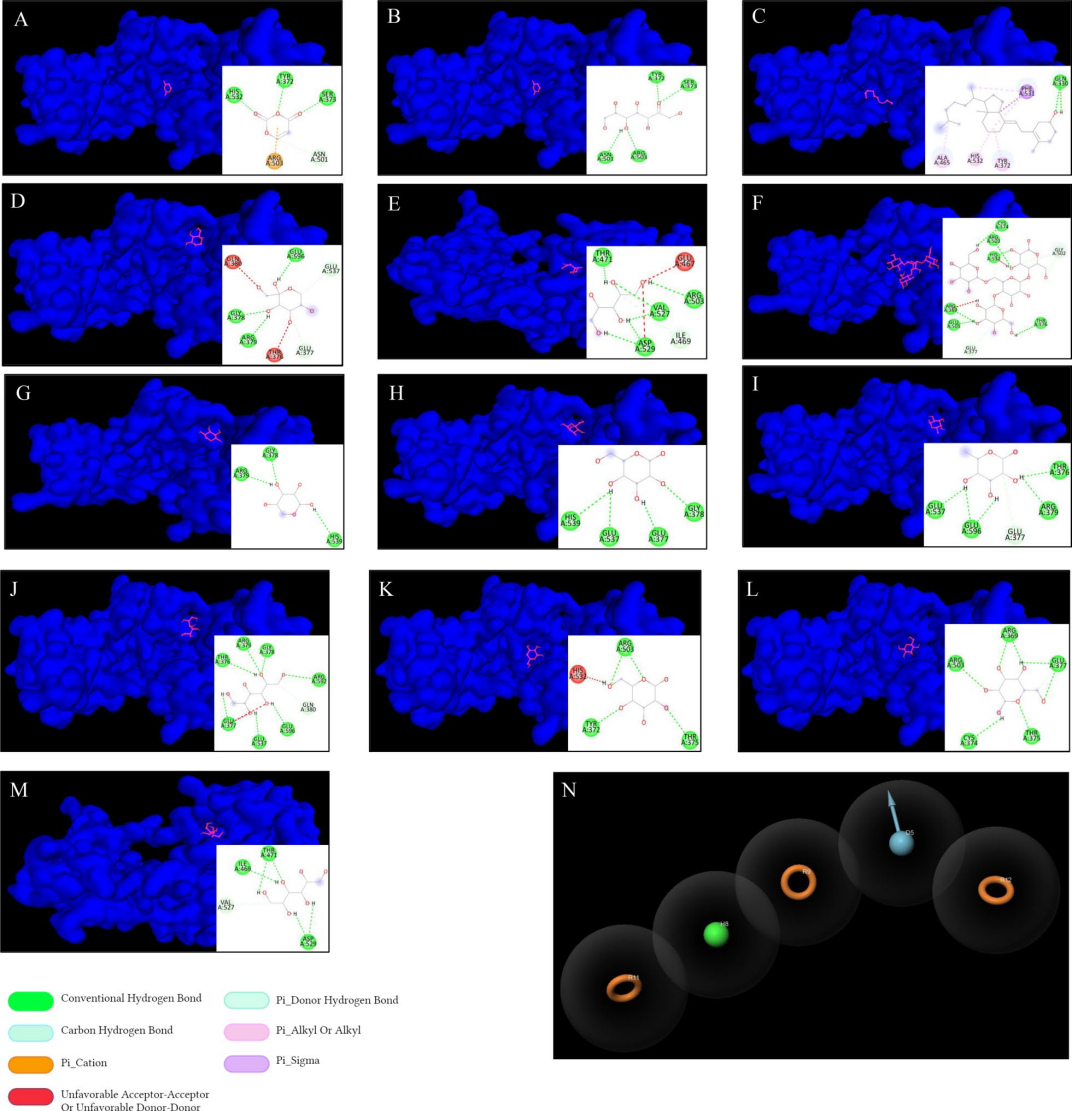
Virtual Screening & Pharmacophore Modeling. **A-M** The chemoinformatics analysis consequences designated the catalog of the binding affinity of bioactive components derived from sparassis latifolia, which targets FN1 macromolecules based on molecular docking analysis in the PyRx offline platform. Binding affinity elaborates the binding energy, and the RMSD score elaborates the stability of this binding energy. Further, the bond type is specified based on PyMOL and BIOVIA Discovery Studio Visualizer software. Different colors in the guidance characterize bond types. **N** The DHRRR pharmacophore model obtained the maximum survival score (5.530872) using 4 authorized ligands for FN1. Bioactive compounds derived from sparassis latifolia did not align with the pharmacophore model based on pharmacophore screening

Upon careful literature review, we have found 13 chemicals categorized as bioactive components derived from sparassis latifolia. These substances have the potential to function as adjunctive therapies for enhancing the efficacy of chemotherapy and alleviating symptoms associated with colorectal cancer. The molecular docking method was employed to compute the binding affinity between bioactive compounds and the cut-point protein. The results indicated that glycogen, mannitol, sorbitol, arabinitol, glucose, galactose, mannose, xylose, fructose, fucose, sedoheptulose, and β–glucans exhibited a favorable affinity when bound to FN1 protein. Therefore, ingesting sparassis latifolia extract, which contains these bioactive substances, may enhance the efficacy of chemotherapy, boost the immune system's response, and reduce inflammation-induced damage in individuals with colorectal cancer. This improvement is achieved by modifying the molecular signaling pathways contributing to colorectal cancer development and altering the expression patterns of genes associated with healthy physiological states. The results of the molecular docking study are indicated in Fig. 7A–M. Further, based on the chemoinformatic analysis, the binding site of bioactive compounds derived from sparassis latifolia over the macromolecule FN1 is visualized in Fig. 7A–M.

The DHRRR pharmacophore model had the highest survival score (5.530872) among the four permitted ligands for FN1 in the binding database library (Fig. 7N). In the present model, the symbol "R" denotes a ring atom, the symbol "D" denotes a donor atom, whereas the symbol "H" represents a hydrogen atom. The ligand's library was screened to determine its alignment with the pharmacophore model. in this study, we found that bioactive compounds derived from sparassis latifolia did not align with the pharmacophore model based on pharmacophore screening (Fig. 7N).

### Effect of the bioactive compounds of *Sparassis latifolia* along with exercise training on the long intestinal phenotype and gene expression biomarkers in the chemotherapy-treated COL mice

**Fig. 8:**
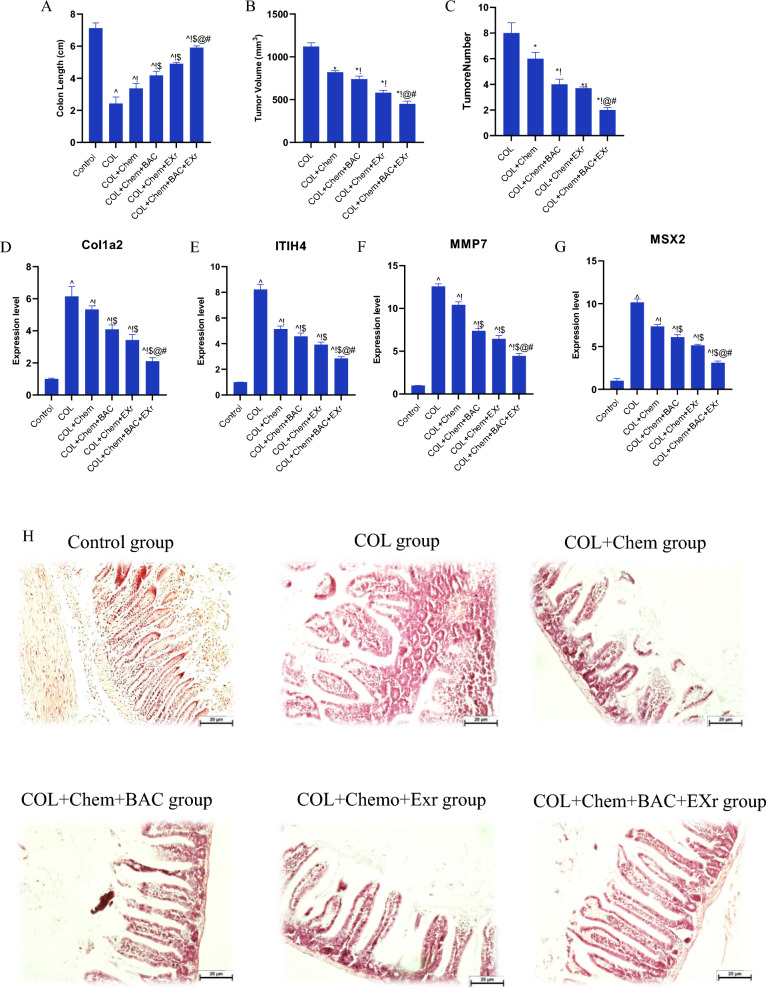
Phenotype and gene expression biomarkers related to colorectal cancer. **A** colon length (cm), **B** tumor size (mm.^3^), **C** tumor number, **D** Relative expression of Col1a2 gene, **E** Relative expression of ITIH4 gene, **F** Relative expression of MMP7 gene, **G** Relative expression of MSX2 gene, **H** The histologic analysis of colon tissue. Scale bars: 40 µm. (^ Demonstrates a significant difference with the control group at p < 0.05, ! Demonstrates significant difference with COL group at p < 0.05, $ Demonstrates significant difference with the COL + Chem group at p < 0.05, @ Demonstrates significant difference with COL + Chem + BAC group at p < 0.05, # Demonstrates significant difference with COL + Chemo + EXr group at p < 0.05)

To evaluate the effect of the bioactive compounds of sparassis latifolia and exercise training on the phenotype feature of colon tissue in the colorectal cancer condition, the colon length (cm), tumor size (mm^3^), tumor number, the and histologic categorization of the colon tissue were assessed. Moreover, we detected the alternation of the vital biomarkers involved in colorectal cancer and bioactive compounds of sparassis latifolia along with exercise training (Fig. 8A–H). Hence, we found that the colon length significantly decreased in the COL group compared with the Normal group (Fig. 8A). We indicated that compared with the COL + Chem group, bioactive compounds of sparassis latifolia and exercise training significantly reduced the colon length, tumor size, and tumor number in chemotherapy-treated COL mice were gavaged bioactive compounds of sparassis latifolia (COL + Chem + BAC group) and chemotherapy-treated COL mice were treated to exercise training with an intensity of low to moderate on the treadmill (COL + Chemo + EXr) (Fig. 8A–C). Notably, the thickness, shortening, and colon adhesion of the mice in the bioactive compounds of sparassis latifolia and exercise training were significantly relieved (Fig. 8A–C). Based on these data, we found that the phenotype features of the colon tissue were improved by bioactive compounds of sparassis latifolia and exercise training.

Growing evidence has indicated that the genes of Col1a2, ITIH4, MMP7, and MSX2 profiles could be critical markers for identifying colorectal cancer. In this study, we explored that the relative expression of the Col1a2, ITIH4, MMP7, and MSX2 are increased in the mice who induced colorectal cancer (the COL group) compared with the Normal group (Fig. 8D–G). In addition, these markers are reduced in the chemotherapy-treated COL mice (COL + Chem + BAC group) compared to the COL group (Figs. 8D–G). Notably, based on our results, the expression level of the COL1A2, ITIH4, MMP7, and MSX2 was regulated in the chemotherapy-treated COL mice were gavaged bioactive compounds of sparassis latifolia (COL + Chem + BAC group) and chemotherapy-treated COL mice were treated to exercise training with an intensity of low to moderate on the treadmill for eight weeks (COL + Chemo + EXr) (Fig. 8D–G). Based on our results, compared with the other groups, the expression level of the COL1A2, ITIH4, MMP7, and MSX2 was decreased in the COL + Chem + BAC + EXr group (Fig. 8D–G). Interestingly, our data demonstrated the synergetic effect of the bioactive compounds of sparassis latifolia along with exercise training (the COL + Chem + BAC + EXr group).

Chemotherapy-treated COL mice were treated with bioactive compounds of sparassis latifolia along with exercise training for eight weeks (the COL + Chem + BAC + EXr group).

We evaluated the organization and structure of the colon tissue sections (Fig. 8H). Compared with the Normal group, we demonstrated that the epithelial glands and colonic villi were destroyed in the COL group. Moreover, goblet cells were reduced, and the cell size was different in the COL group than in the Normal group (Fig. 8H). Compared with the COL group, COL + Chem + BAC, and COL + Chemo + EXr groups reversed the organization and structure of the colon tissue (Fig. 8H). Hematoxylin and eosin stain has revealed that the bioactive compounds of sparassis latifolia along with exercise training (COL + Chem + BAC + EXr group) had more effective on the cell structure and organization tissue compared with the other groups (Fig. 8H).

### The inflammation molecules and oxidative stress capacity were regulated by bioactive compounds of sparassis latifolia along with exercise training in the chemotherapy condition

**Fig. 9:**
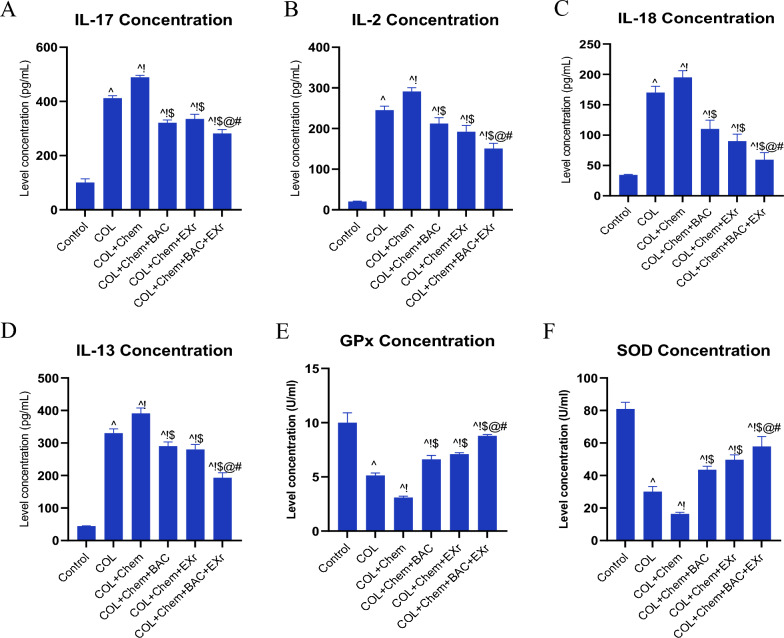
The inflammation factors and oxidative capacity were improved by sparassis latifolia bioactive compounds and exercise training. **A** Concentration of the IL-17 evaluated by ELISA method. **B** Concentration of the IL-2 evaluated by ELISA method. **C** Concentration of the IL-18 evaluated by ELISA method. Concentration of the IL-13 evaluated by ELISA method. **E** Concentration of the GPx was evaluated using the ELISA method. **F**. Concentration of the SOD evaluated by ELISA method. (^ Demonstrates a significant difference with the control group at p < 0.05, ! Demonstrates significant difference with COL group at p < 0.05, $ Demonstrates significant difference with the COL + Chem group at p < 0.05, @ Demonstrates significant difference with COL + Chem + BAC group at p < 0.05, # Demonstrates significant difference with COL + Chemo + EXr group at p < 0.05)

The concentration of the IL-17, IL-2, IL-18, IL-13, GPx, and SOD were dysregulated in the COL group compared with the Normal group (Fig. 9A–F). The inflammation molecules (IL-17, IL-2, IL-18, and IL-13) were significantly enhanced (Fig. 9A–D), and oxidative stress markers (GPx and SOD) in the COL group were reduced (Fig. 9E–F) in comparison with the Normal group. Compared with the COL group, the chemotherapy treatment negatively affected the inflammation molecules and oxidative stress capacity (Fig. 9A–F). In addition, chemotherapy-treated COL mice were gavaged bioactive compounds of sparassis latifolia (COL + Chem + BAC group) and chemotherapy-treated COL mice were treated to exercise training with an intensity of low to moderate (COL + Chemo + EXr group) regulated the inflammation molecules (IL-17, IL-2, IL-18, and IL-13) and oxidative stress markers (GPx and SOD) (Fig. 9A–F). Interestingly, compared with the other groups, chemotherapy-treated COL mice were treated with bioactive compounds of sparassis latifolia along with exercise training (COL + Chem + BAC + EXr group), which had a positive effect and decreased the chemotherapy treatment adverse effect.

### The effect of the bioactive compounds of sparassis latifolia along with exercise training on the constructed lncRNA-miRNA-mRNA mapping

**Fig. 10:**
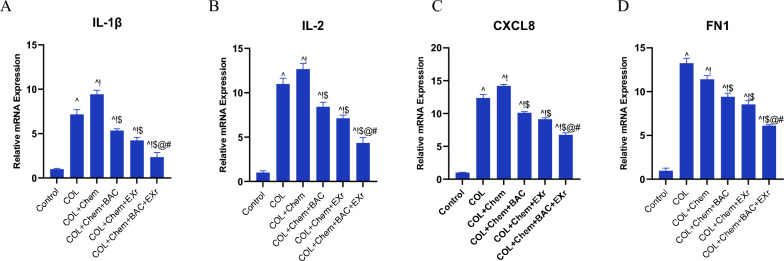
Bioactive compounds of sparassis latifolia along with exercise training alleviated the vital hub genes in the COL mice injected with chemotherapy 5-FU. **A** Relative expression of the IL-1β, **B** Relative expression of the IL-2, **C** Relative expression of the CXCL8, **D**. Relative expression of the FN1. (^ Demonstrates a significant difference with the control group at p < 0.05, ! Demonstrates significant difference with COL group at p < 0.05, $ Demonstrates significant difference with the COL + Chem group at p < 0.05, @ Demonstrates significant difference with COL + Chem + BAC group at p < 0.05, # Demonstrates significant difference with COL + Chemo + EXr group at p < 0.05)

Based on the bioinformatic analysis, we found the constructed competing endogenous RNAs (ceRNAs) mapping involved in colorectal cancer. Based on these data, we selected the vital small molecules related to colorectal cancer. Moreover, we suggested that this constructed lncRNA-miRNA-mRNA has potential therapeutic and management targets. Hence, based on the artificial analysis, we detected critical genes involved in colorectal cancer, including IL-1β, IL-2, CXCL8, and FN1. Our data indicated that the relative expression of the IL-1β, IL-2, CXCL8, and FN1 was significantly enhanced in the colorectal tissue of the COL group compared with the control group (Fig. 10A–D). In addition, we explored that the expression gene profiles were increased by treating the chemotherapy (5-FU, 150 mg/kg, b.w.) in the COL + Chem group compared with the COL group. Interestingly, we found that the consumption of the bioactive compounds of sparassis latifolia (COL + Chem + BAC group) and mice were treated to exercise training with an intensity of low to moderate on the treadmill (COL + Chemo + EXr group) reversed the relative expression of the IL-1β, IL-2, CXCL8, and FN1 compared with COL + Chem group (Fig. 10A–D). Moreover, the synergetic effect of the bioactive compounds of sparassis latifolia, along with exercise training, predominantly reduced the gene expression of the IL-1β, IL-2, CXCL8, and FN1 in comparison of the other groups (Fig. 10A–D).

**Fig. 11:**
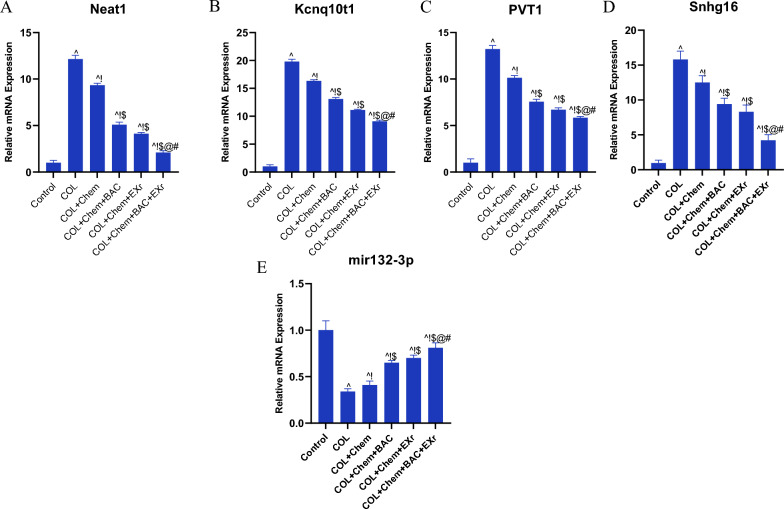
Bioactive compounds of sparassis latifolia and exercise training regulated the ceRNAs network. **A** Expression level of the Neat1, **B** Expression level of the Kcnq1ot1, **C** Expression level of the PVT1, **D** Expression level of the Snhg16, **E** Expression level of the miR132-3p. ^ Demonstrates a significant difference with the control group at p < 0.05, ! Demonstrates significant difference with COL group at p < 0.05, $ Demonstrates significant difference with the COL + Chem group at p < 0.05, @ Demonstrates significant difference with COL + Chem + BAC group at p < 0.05, # Demonstrates significant difference with COL + Chemo + EXr group at p < 0.05

Furthermore, the lncRNA-miRNA was predicated based on the in-silico analysis. Hence, we found that the Neat1, Kcnq1ot1, PVT1, and Snhg16 could bind to the miR132-3p. Moreover, miR132-3p might target the FN1 in colorectal cancer. Based on our results, the relative expression of the Neat1, Kcnq1ot1, PVT1, and Snhg16 was overexpressed in the COL group compared with the control group (Fig. 11A–D). Notably, our data revealed that the chemotherapy (5-FU, 150 mg/kg, b.w.) could dysregulate the expression level of the Neat1, PVT1, Kcnq1ot1, and Snhg16 in the COL + Chem group compared with the control and COL groups (Fig. 11A–D). Interestingly, we suggested that the bioactive compounds of sparassis latifolia could affect the Neat1, PVT1, Kcnq1ot1, and Snhg16 expression and modify the binding affinity of the lncRNA-miRNA. Moreover, exercise training could ameliorate the Neat1, PVT1, Kcnq1ot1, and Snhg16 expression levels compared with other groups (Fig. 11A–D). It should be noted that the expression level of the Neat1, PVT1, Kcnq1ot1, and Snhg16 was regulated in the COL + Chem + BAC + EXr group (Fig. 11A–D).

In this study, the expression level of the miR132-3p was assessed (Fig. 11E). Based on our results, the relative expression of the miR132-3p was significantly reduced in the COL group compared with the control group (Fig. 11E). Furthermore, exercise training bioactive compounds of sparassis latifolia could regulate the expression of the miR132-3p compared with the COL group (Fig. 11E). Interestingly, data has indicated the synergetic effect on the expression of the miR132-3p in the COL + Chem + BAC + EXr group compared with other groups (Fig. 11E).

### Correlation between lncRNAs-miRNA with FN1 as critical genes in the network

**Fig. 12:**
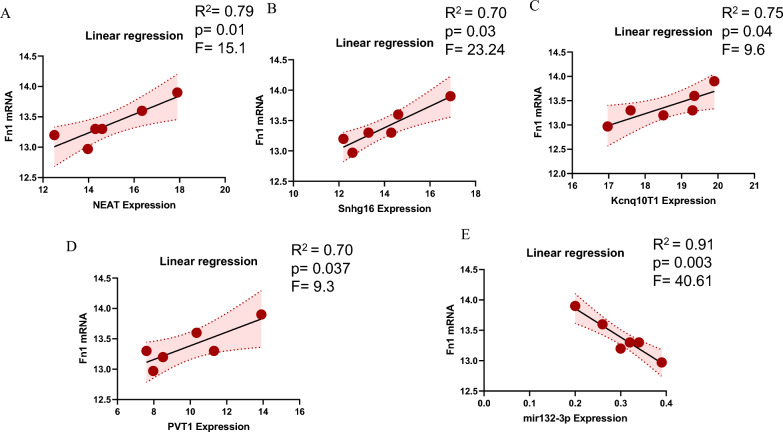
Correlation between lncRNAs-miRNA-mRNA mapping. **A** Correlation analysis of FN1 mRNA with NEAT. **B** Correlation analysis of FN1 mRNA with Snhg16. **C** Correlation analysis of FN1 mRNA with Kcnq1ot1. **D** Correlation analysis of FN1 mRNA with PVT1. **E**. Correlation analysis of FN1 mRNA with miR132-3p

The relationship between lncRNAs-miRNA with FN1 was estimated by Pearson correlation (Fig. 12A–E). Our results have demonstrated that the expression level of the Neat1, PVT1, Kcnq1ot1, and Snhg16 had a positive association with the relative expression of FN1 (Fig. 12A–D). Hence, upregulation of the Neat1, PVT1, Kcnq1ot1, and Snhg16 were associated with the dysregulation of the FN1. Moreover, we analyzed the correlation between miR132-3p and FN1. Based on the Fig. 12E, miR132-3p had a negative correlation with the relative expression of FN1.

## Discussion

In the current study, we evaluated complementary medicine of the bioactive compounds of Sparassis latifolia and exercise training against colorectal cancer induced by the combination of DSS and AOM in the chemotherapy (5-FU) condition. The concurrent utilization of DSS and AOM has been observed to elicit the development of inflammatory colon cancer by a notable reduction in colon length and facilitation of colorectal adenoma proliferation.

The gene profiles (Col1a2, ITIH4, MMP7, and MSX2) were identified as critical markers in colorectal cancer. In addition, the inflammation molecules (IL-17, IL-2, IL-18, and IL-13) and oxidative stress capacity (GPx and SOD) were dysregulated in colorectal cancer as well as administration of 5-FU. Notably, we conducted a bioinformatic analysis and discovered the critical hub genes involved in colorectal cancer. Based on the *In-silico* analysis, we explored the construction of the lncRNA-miRNA-mRNA mapping dysregulated in colorectal cancer and administration of 5-FU. Hence, the relative expression of the IL-1β, IL-2, CXCL8, and FN1 was overexpression by colorectal cancer and administration of 5-FU. Interestingly, we predicted the lncRNA-miRNA via the *In-silico* analysis. Therefore, we discovered the Neat1, Kcnq1ot1, PVT1, and Snhg16 (as lncRNAs) could bind to the miR132-3p. Furthermore, miR132-3p could target the FN1 in colorectal cancer. These markers were dysregulated in the colorectal cancer and administration of 5-FU.

In this study, we demonstrated that the administration of 5-FU had adverse effects. Our results indicated that administration of 5-Fluorouracil (5-Fu) could enhance the inflammatory agents, decrease the oxidative stress capacity, and critical markers in colorectal cancer, although the colon length (cm), tumor size (mm^3^), tumor number, and histologic categorization of the colon tissue were improved. These data were in accordance with previous evidence. Moreover, administration of 5-FU (150 mg/kg, b.w.) dysregulated the expression gene profiles (IL-1β, IL-2, CXCL8, and FN1) and lncRNA-miRNA mapping.

5-Fu is widely employed as an antimetabolite drug in the management of several malignancies, including breast, colorectal, gastric, and head and neck cancers [76, 77]. This preference stems from its extensive range of antitumor effects and its ability to enhance the efficacy of other anticancer treatments through synergistic interactions [77]. The anticancer activity of the substance is attributed to its ability to impede the processes of DNA and RNA production [77]. Multiple studies have indicated that cardiotoxicity induced by 5-Fu is facilitated by generating reactive oxygen species (ROS), resulting in oxidative stress [77, 78]. This oxidative stress then triggers the activation of various inflammatory and apoptotic pathways [78]. This study examines the impact of administering 5-FU on oxidative stress by stimulating the generation of reactive oxygen species (ROS) and reducing the activities of antioxidant enzymes. The administration of 5-FU has been extensively associated with promoting intracellular oxidative stress, characterized by the rapid production of significant quantities of protein carbonyls, free radicals, and lipid peroxidation [78].

Furthermore, 5-FU has the ability to modify the balance of free iron and Ca2^+^ within cells by forming complexes that increase redox potentials [79]. This alteration ultimately results in the production of hydrogen peroxide (H_2_O_2_) and ROS. Oxidative events induce changes in cellular macromolecules, leading to subsequent cellular death [79].

Based on our data, bioactive compounds of Sparassis latifolia along with exercise training, improved the colon length (cm), tumor size (mm^3^), tumor number, and histologic categorization of the colon tissue in the chemotherapy-treated COL mice. In addition, the expression levels of the Col1a2, ITIH4, MMP7, and MSX2 were regulated in chemotherapy-treated COL mice treated with bioactive compounds of Sparassis latifolia and exercise training for eight weeks.

Exercise can increase motivation to change lifestyle behaviors, improve aerobic fitness and physical performance, control fatigue, and increase quality of life. As a non-pharmacological intervention and preventive measure, exercise also helps reduce the risk of cancer [80]. More importantly, exercise training has been implicated in various cancers during cancer treatment [81]. Exercise and physical activity have many processes that contribute to their ability to prevent and treat cancer [82]. The regulation of proliferative signaling pathways is a crucial process [82]. Interfering with the signaling pathways that promote cell growth decreases the likelihood of cells becoming cancerous [82].

Consumption of the bioactive compounds of sparassis latifolia and mice were treated to exercise training with an intensity of low to moderate reversed expression level of the IL-1β, IL-2, CXCL8, and FN1. Furthermore, the synergetic effect of the bioactive compounds of sparassis latifolia, along with exercise training, mitigated the gene expression of the IL-1β, IL-2, CXCL8, and FN1. Moreover, our data demonstrated the synergetic effect of the bioactive compounds of sparassis latifolia along with exercise training. Interestingly, we revealed that the bioactive compounds of sparassis latifolia could ameliorate the Neat1, PVT1, Kcnq1ot1, and Snhg16 expression and modify the binding affinity of the lncRNA-miRNA. Furthermore, exercise training regulated the expression level of Neat1, PVT1, Kcnq1ot1, and Snhg16.

The desire to use herbal and natural products or their derivatives to reduce the side effects caused by chemotherapy or to increase the sensitivity of tumor cells to chemotherapy agents (chemical sensitizers) has attracted much attention in the last few decades [6]. Sparassis latifolia mushroom is a rare edible medicinal mushroom with a fruit body, rich nutritional value, and many effective substances [30]. Asparagus latifolia polysaccharides (SLPs) are β-glucan-rich edible fungal polysaccharides and are an essential nutritive component in Asparagus latifolia, which has been reported to have antitumor effects in mice with high vasodilation and hemorrhagic reactions [28]. Oral administration of β-glucan from Sparassis latifolia may modulate cytokines in the spleen by activating Peyer's patches [30]. SLPs have antitumor effects by regulating the expression of TLR4 and MyD88 in the small intestine and increasing the production of TNF-α induced by resistance exercise. However, its effects on gastrointestinal cancer are rarely reported [30, 83]. Many reports showed that edible mushrooms of the genus Asparagus are rich in protein, vitamins, polysaccharides, etc. [83]. As the main active macromolecules, polysaccharides show various biological activities, including immune modulation, antitumor, and antioxidant effects [84]. It is well known that the biological activity of polysaccharides is closely related to their structure, so the structural analysis of polysaccharides is the basis of studying their biological activity. Sparassis latifolia contained high levels of β-glucan, which was found to be about 40% [84].

Multiple research investigations have demonstrated the potential of polysaccharides in promoting intestinal health, alleviating chronic inflammation-induced intestinal damage, and enhancing the differentiation degree of colon intramucosal cancer [84].

The prerequisite for palliative care in the primary setting demands the readiness of health care professionals to communicate coherently and empathetically with the patient. Primary palliative care usually focuses on delineating realistic and achievable treatment goals, facilitating patient choice by providing adequate information, and assessing patient values and preferences to advance care planning [85]. The inherent belief is that symptoms can be prevented or easily managed with early treatment, improving the patient's quality of life [86]. Most treatments include education, evidence-based methods used to control symptoms, and psychosocial support [86]. In principle, primary palliative care is based on a preventive approach and is usually offered to patients without high symptom burden or unmet psychosocial needs. One of the common side effects in cancer patients who undergo long-term chemotherapy and radiotherapy is cardiovascular complications [85].

As CRC is a progressive multifactorial lifestyle disease, the pathophysiological mechanism and critical genes involved in tumor growth and progression remain unknown. Recent research shows that lncRNAs generally last longer than 200 nucleotides, are more stable in biological tissues and fluids than protein-coding genes, and have tissue-specific expression patterns. Growing evidence suggests that lncRNAs influence gene expression via unique molecular pathways. In several cancer types, aberrant lncRNAs expression is disturbed, which affects symptoms and consequences [87].

MicroRNAs (miRNAs) are a kind of conservatively structured non-coding gene sequences found in many eukaryotic species [88]. More than typical miRNAs are now involved in cell carcinogenesis [89, 90]. These miRNAs are situated at fragile chromosomal locations. Research has shown that increasing levels of miR-132, which comprises miR-132-3p and miR-132-5p, may reduce the incidence of certain cancers [91, 92]. Overexpression of miR-132-3p, as shown by Su Y et al., prevents the growth of lung adenocarcinoma cells and enhances cell death via the inhibition of pGSK-3β and β-catenin expression (93). According to research by Li et al., the overexpression of miR-132-3p in breast cancer regulates the Notch signaling system, affecting the self-renewal capacity of tumor stem cells in breast cancer [94]. The precise regulatory mechanism likely involves controlling the ESR1-induced downregulation of Let7b expression, which in turn causes the release of Numb and, eventually, inhibits the activation of the Notch signaling pathway [94]. Patients with low-expression levels of miR-132-3p in the serum had a poor prognosis, and Han S et al. confirmed that low expression of miR-132-3p was an independent risk factor affecting overall survival (OS) [95]. They achieved this by comparing the serum expression levels of miR-132-3p in 92 patients with renal carcinoma cancer who underwent chemotherapy. The findings above provide evidence that miR-132-3p is involved in carcinogenesis and cancer development; however, no studies involving miR-132-3p in colon cancer have been found in the literature. miR-132-3p suppresses the growth, progression, and invasion of colorectal cancerous cells [96]. miR-132-3p is most probably accomplished by downregulating the specific expression of FOXP2. This position can potentially be further investigated for therapeutic applications in CRC [97]. Previous studies have demonstrated that miR-132-3p regulates cell proliferation, metastasis, and migration in colorectal cancer (CRC) by interacting with CREB5 [98, 99]. We found that the expression of the miR132-3p was decreased in the COL group compared with the control group. Furthermore, exercise training bioactive compounds of sparassis latifolia could regulate the expression of the miR132-3p compared with the COL group. Interestingly, data indicated the synergetic effect on the expression of the miR132-3p in the COL + Chem + BAC + EXr group compared with other groups.

## Conclusion

Significant advancements have been made in the treatment of COL. However, the prognosis for patients is still unsatisfactory owing to constraints such as multi-drug resistance and tumor recurrence. Consequently, there has been an increased focus on alternative treatments and the use of supplements. Furthermore, the identified hub gene and lncRNA, together with the diagnostic and prognostic indicators, have the potential to serve as druggable proteins for innovative therapeutic targeting of COL.

## Data Availability

All of the raw data and the rest of the materials are remained in of Islamic Azad University—Isfahan (Khorasgan) Branch and are available upon request.
